# Diffusion-weighted imaging as predictor of acute ischemic stroke etiology

**DOI:** 10.1590/0004-282X-ANP-2021-0080

**Published:** 2022-02-28

**Authors:** Alejandro Michel Brunser, Eloy Mansilla, Victor Navia, Enrico Mazzon, Alexis Rojo, Gabriel Cavada, Verónica Olavarría, Paula Muñoz Venturelli, Pablo Manuel. Lavados

**Affiliations:** 1 Clínica Alemana de Santiago, Department of Neurology and Psychiatry, Vascular Neurology Service, Neurology Unit, Santiago, Chile.; 2 Clínica Alemana de Santiago, Emergency Department, Vascular Neurology Service, Neurology Unit, Santiago, Chile.; 3 Universidad del Desarrollo, Facultad de Medicina Clínica Alemana, Santiago, Chile.; 4 Clínica Alemana de Santiago, Department of Critical Care, Santiago, Chile.; 5 Hospital Clínico Herminda Martin de Chillán, Servicio de Salud Ñuble, Servicio de Neurología, Chillán, Chile.; 6 Clínica Alemana de Santiago, Unidad de Investigación y Ensayos Clínicos, Departamento de Desarrollo Acedémico e Investigación, Santiago, Chile.; 7 Universidad del Desarrollo, Facultad de Medicina Clínica Alemana Santiago, Instituto de Ciencias e Innovación en Medicina, Centro de Estudios Clínicos, Santiago, Chile.

**Keywords:** Stroke, Ischemic Stroke, Diffusion Magnetic Resonance Imaging, Diagnosis, Accidente Cerebrovascular, Accidente Cerebrovascular Isquémico, Imagen de Difusión por Resonancia Magnética, Diagnóstico

## Abstract

**Background:**

Topographic patterns may correlate with causes of ischemic stroke.

**Objective:**

To investigate the association between diffusion-weighted imaging (DWI) and Trial of Org 10172 in Acute Stroke Treatment (TOAST) classification.

**Methods:**

We included 1019 ischemic stroke patients. DWI were classified as: i) negative; ii) DWI single lesion (cortico-subcortical, cortical, subcortical ≥20 mm, or subcortical <20 mm); iii) scattered lesions in one territory (small scattered lesions or confluent with additional lesions); and iv) multiple lesions (multiple unilateral anterior circulation [MAC], multiple posterior circulation [MPC], multiple bilateral anterior circulation [MBAC], and multiple anterior and posterior circulations [MAP]).

**Results:**

There was a relationship between DWI patterns and TOAST classification (p<0.001). Large artery atherosclerosis was associated with small, scattered lesions in one vascular territory (*Odds Ratio* [OR] 4.22, 95% confidence interval [95%CI] 2.61–6.8), MPC (OR 3.52; 95%CI 1.54–8.03), and subcortical lesions <20 mm (OR 3.47; 95%CI 1.76–6.85). Cardioembolic strokes correlated with MAP (OR 4.3; 95%CI 1.64–11.2), cortico-subcortical lesions (OR 3.24; 95%CI 1.9–5.5) and negative DWI (OR 2.46; 95%CI 1.1–5.49). Cryptogenic strokes correlated with negative DWI (OR 4.1; 95%CI 1,84–8.69), cortical strokes (OR 3.3; 95%CI 1.25–8.8), MAP (OR 3.33; 95%CI 1.25–8.81) and subcortical lesion ≥20 mm (OR 2.44; 95%CI 1,04–5.73). Lacunar strokes correlated with subcortical lesions diameter <20 mm (OR 42.9; 95%CI 22.7–81.1) and negative DWI (OR 8.87; 95%CI 4.03–19.5). Finally, MBAC (OR 9.25; 95%CI 1.12–76.2), MAP (OR 5.54; 95%CI 1.94–15.1), and MPC (OR 3.61; 95%CI 1.5–8.7) correlated with stroke of other etiologies.

**Conclusions:**

A relationship exists between DWI and stroke subtype.

## RESUMEN

**Antecedentes:**

Los patrones topográficos pueden correlacionarse con las causas del accidente cerebrovascular isquémico.

**Objetivo:**

Investigar la asociación entre imágenes ponderadas por difusión por resonancia nuclear magnética (dRNM) y el ensayo de Org 10172 en la clasificación de tratamiento agudo de accidentes cerebrovasculares (TOAST).

**Métodos:**

Fueron incluidos 1.019 pacientes con accidente cerebrovascular isquémico. Las dRNM fueron clasificadas como: i) negativa; ii) dRNM lesión única (cortico-subcortical, cortical, subcortical ≥20 mm, o subcortical <20 mm); iii) lesiones disgregadas un territorio vascular (pequeñas lesiones dispersas o confluentes con lesiones adicionales); y iv) lesiones múltiples (unilaterales de circulación anterior [MAC], de circulación posterior [MPC], bilaterales de circulación anterior [MBAC] y de circulación anterior y posterior [MAP]).

**Resultados:**

Existió relación entre los patrones de dRNM y la clasificación TOAST (p<0,001). La aterosclerosis de las arterias grandes se asoció con lesiones pequeñas y disgregadas en un territorio vascular (*Odds Ratio* [OR] 4,22, intervalo de confianza del 95% [IC95%] 2,61–6,8), MPC (OR 3,52; IC95% 1,54–8,03), y lesiones subcorticales <20 mm (OR 3,47; IC95% 1,76–6,85). Cardioembolias se relacionaron con MAP (OR 4,3; IC95% 1,64–11,2), lesiones cortico-subcorticales (OR 3,24; IC95% 1,9–5,5) y dRNM negativas (OR 2,46; IC95% 1,1–5,49). Los accidentes cerebrovasculares criptogénicos se relacionaron con dRNM negativas (OR 4,1; IC95% 1,84–8,69), accidentes cerebrovasculares corticales (OR 3,3; IC95% 1,25–8,8), MAP (OR 3,33; IC95% 1,25–8,81) y lesiones subcorticales ≥20 mm (OR 2,44; IC95% 1,04–5,73). Los accidentes cerebrovasculares lacunares se correlacionaron con lesiones subcorticales de diámetro <20 mm (OR 42,9; IC95% 22,7–81,1) y dRNM negativas (OR 8,87; IC95% 4,03–19,5). Finalmente, MBAC (OR 9,25; IC95% 1,12–76,2), MAP (OR 5,54; IC95% 1,94–15,1) y MPC (OR 3,61; IC95% 1,5–8,7) se relacionaron con accidentes cerebrovasculares de otras etiologías.

**Conclusiones:**

Existe relación entre dRNM y subtipo de accidente cerebrovascular.

## INTRODUCTION

A precise and early diagnosis of acute ischemic stroke (AIS) etiologic subtype is important for therapeutic decisions that may influence stroke recurrence, management, and prognosis^1,2^. The most frequently used method for causative subtype classification is the Trial of Org 10172 in Acute Stroke Treatment (TOAST), which divides AIS into subtypes, based primarily on infarction mechanism^1,2^.

Diffusion-weighted imaging (DWI) is sensitive and specific for the early detection of hyper-acute ischemic lesions, even those of very small size^3–5^. Ischemic lesion topography on DWI may correlate with stroke subtypes. However, previous studies demonstrating this association were limited to specific stroke etiologies or DWI patterns, included transient ischemic attacks, were retrospective, included AIS of the anterior circulation only, or did not consider negative DWI results^5–13^, which could represent a proportion as high as 13% of the AIS admitted to the emergency room (ER)^4^.

In this study we aimed to determine the association between DWI ischemic topography and AIS stroke subtype using the causative TOAST classification. A large prospective cohort of consecutive, unselected patients with AIS admitted to out center was evaluated.

## METHODS

In this prospective study, patients with AIS admitted to the ER between December 2012 and June 2019 were evaluated by the neurologist on call. Age, stroke risk factors, and time from stroke onset to arrival to the ER, defined as the last time at which the patient was known to be free of any neurological deficits, were recorded. Stroke severity was assessed with the National Institutes of Health Stroke Scale (NIHSS). Patients were then studied with the local neuroimaging protocol, which has been described previously^4^. The protocol consists of a non-contrast brain computed tomography (NCCT) and, in patients without contraindication, an immediate spiral computed tomographic angiography to evaluate the cervical and intracranial arteries (CTA) and DWI-MRI. If a CTA could not be obtained, an acute magnetic resonance angiography (AngioMR) was performed.

The time from symptom onset to the time when the DWI was performed was recorded, as well as the model of the MRI equipment in which this study was carried out. Evaluations were performed either in a GE HdxT 1.5-T MRI or in a Siemens Skyra 3-Tesla and varied according to their availability upon arrival. A standardized protocol was applied including axial acquisitions and, in patients with negative results, thin coronal sections were also performed^4^.

After the initial evaluation, most patients were transferred to the Stroke Unit for at least a 48-hour follow up. Telemetry monitoring was performed in all cases, and as soon as possible cardiologists carried out a transthoracic echocardiogram. Additional evaluations were performed as required to determine infrequent causes of AIS.

Patients with negative baseline DWI results in the ER were re-evaluated after 24 hours with either brain NCCT or MRI according to the preference of the attending neurologist.

Patients with repeated negative imaging, but with an evident neurovascular syndrome, and no other alternative diagnosis explaining the patient's initial symptoms after extensive workup were finally diagnosed as stroke^3^.

**Figure 1:**
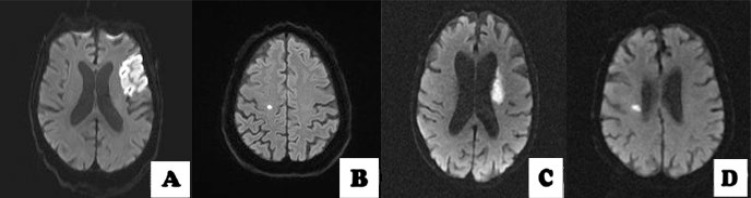
(A) cortico-subcortical lesion; (B): pure cortical lesion; (C) subcortical lesion with a diameter ≥20 mm; (D) subcortical lesion with a diameter <20 mm.

**Figure 2:**
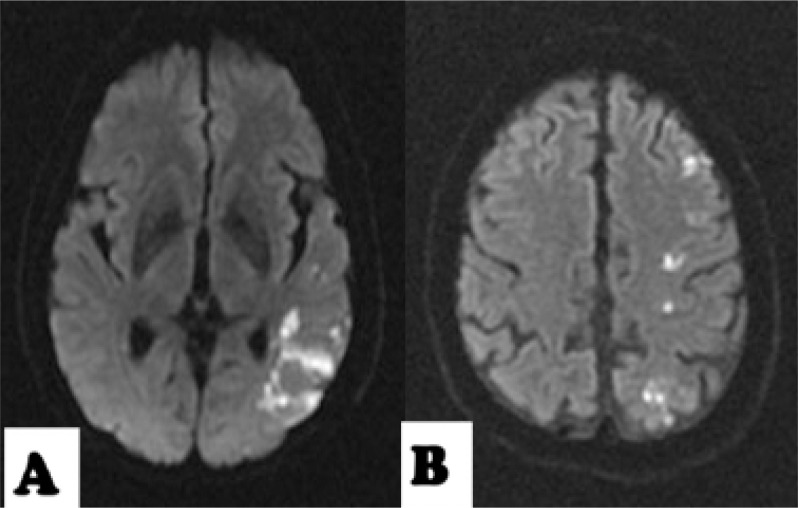
Scattered lesions in one vascular territory. A: scattered lesions or confluent lesions greater than 15 mm with an additional lesion. B: Scattered lesions in one vascular territory. A: small ones, less than 15 mm.

**Figure 3:**
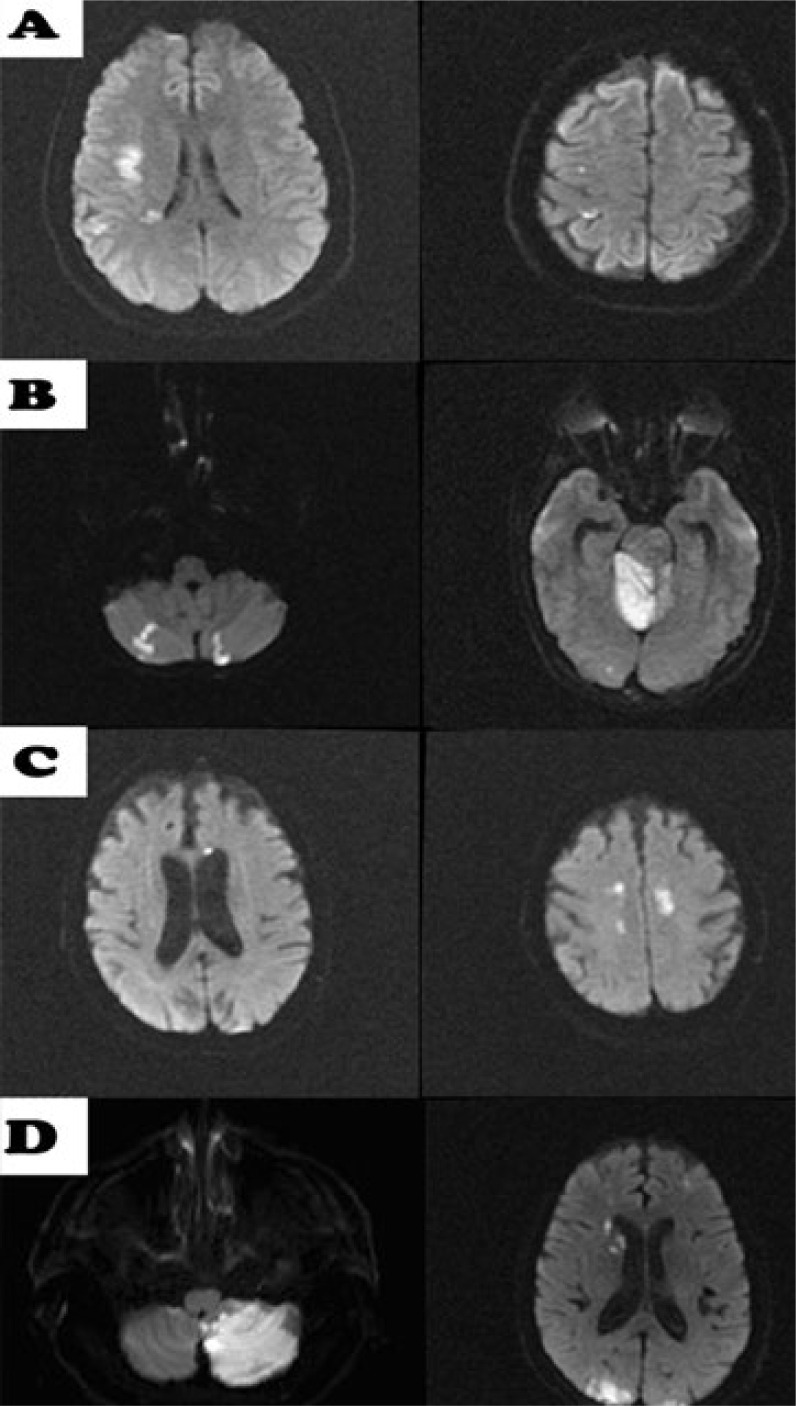
Multiple lesions in multiple vascular territories. (A) unilateral anterior circulation; (B) posterior circulation; (C) bilateral anterior circulations; (D) anterior and posterior circulations.

DWI topography of the ischemic lesions was determined by an experienced stroke neurologist (AB), based on a modification of a previous classification^10^. In this article, we included negative baseline DWI results as a variable and we changed the subgroup criteria for single subcortical lesions from 15 mm to 20 mm. Ischemic baseline patterns were classified as follows: i) absence of acute lesion or normal baseline DWI; ii) single lesions group (which includes as subgroups cortico-subcortical lesion, pure cortical lesion, subcortical lesion with a diameter ≥20 mm or subcortical lesions with a diameter <20 mm) (Figure 1); iii) scattered lesions of less than 15 mm in one vascular territory (SLVT) and scattered and confluent lesions of 15 mm or more (Figure 2); and iv) multiple lesions in multiple vascular territories (unilateral anterior circulation [MAC], posterior circulation [MPC], bilateral anterior circulation [MBAC], or both anterior and posterior circulation [MAP]) (Figure 3).

The reviewer was blinded to the clinical data of patients; the etiological stroke classification was made at the time of discharge by the treating neurologist, and according to the TOAST classification^2^.

The Ethics Committee of Universidad del Desarrollo, Clínica Alemana de Santiago approved the protocol, and the patients or their relatives provided written informed consent as part of the local prospective stroke registry.

### Statistical analysis

DWI patterns and their association with stroke etiology were described in an analysis of simple correspondence to identify closeness between the pattern and the etiology. The significance of this proximity was evaluated with Fisher's exact test. The strength of the association was calculated as *Odds Ratio* (OR) with a level of significance of 5% and 95% confidence interval (95%CI).

The significant associations are shown in a bi-plot obtained through a simple correspondence analysis.

Data were processed with the Stata v 14.0 software.

## RESULTS

During the study period, 1108 consecutive AIS patients were admitted to the ER; 1019 (91.9%) were included in this analysis. Eighty-nine patients were excluded because of a contraindication for MRI (peacemaker, agitation, critical medical condition, or low quality of the DWI image).

Mean patient age was 72.5±17.4 years and 562 (55.5%) patients were women. Mean NIHSS was 3±7.1. Mean time from stroke onset to ER arrival was 322±1786 minutes and mean time from AIS onset to DWI was 376±1803 minutes. In relation to time to imaging, 450 (44.2%) patients were assessed with DWI within 4.5 hours of symptom onset and in 824 (80.9%) DWI was performed within the first 24 hours; 97 cases (9.5%) were evaluated between 24 and 48 hours and only 98 (9.6%) patients were assessed with DWI more than 48 hours after symptom onset. A 3T MRI was used in 193 (18.9%) of the cases, and 308 (30.2%) patients were treated with intravenous thrombolysis.

Ischemic lesion patterns in baseline DWI by the TOAST classification are described in Table 1. No lesion was found in baseline DWI evaluation of 142 patients (14%); a single lesion was observed in 483 patients (47.3%), scattered lesions in one vascular territory were observed in 198 cases (19.4%). Finally, multiple lesions in multiple vascular territories were found in 196 patients (19.3%).

**Table 1:** Ischemic lesion patterns at baseline diffusion-weighted imaging by Trial of Org 10172 in Acute Stroke Treatment classification.

DWI pattern/TOAST subtypes		LAA (n=173)	Cardioembolic (n=282)	Cryptogenic (n=289)	Lacunar (n=153)	Other causes (n=122)	Total (n=1,019)
Negative DWI		8	30	47	46	11	142
Single lesion							483
	Corticosub-cortical	20	84	61	0	18	183
	Cortical	5	18	26	0	5	54
	Subcortical 20 mm and over	7	24	27	1	11	70
	Subcortical less 20 mm	26	16	14	105	15	176
SLVT							198
	SLVT Small (<15 mm)	61	22	33	0	15	131
	SLVT Confluent lesion (≥15 mm)	16	25	20	0	6	67
Multiple lesions							196
	MAC	4	10	5	0	4	23
	MPC	18	9	17	0	13	57
	MBAC	3	12	13	1	7	36
	MAP	5	32	26	0	17	80

DWI: diffusion-weighted imaging; LAA: large-artery atherosclerosis; SLVT: scattered lesions in one vascular territory; MAC: multiple lesions in unilateral anterior circulation; MPC: multiple lesions in posterior circulation; MBAC: multiple lesions in the bilateral anterior circulation; MAP: multiple lesions anterior and posterior circulations.

**Figure 4:**
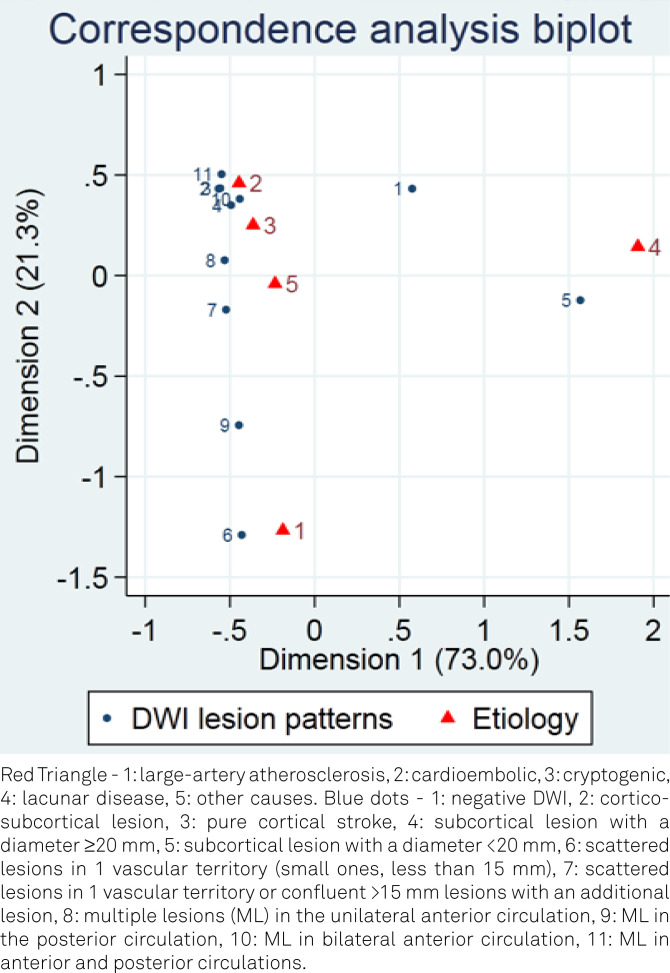
Correspondence analysis biplot between diffusion-weighted imaging lesions patterns and etiology by Trial of Org 10172 in Acute Stroke Treatment posterior circulations.

The assessment of the association between baseline DWI patterns and AIS TOAST subgroups is shown in Figure 4 and Table 2. Large artery atherosclerosis (LAA) was associated with small scattered lesions in one vascular territory (OR 4.22; 95%CI 2.61–6.8), MPC (OR 3.52; 95%CI 1.54–8.03), and with subcortical lesions of less than 20 mm (OR 3.47; 95%CI 1.76–6.85).

**Table 2:** Associations between diffusion-weighted imaging patterns and Trial of Org 10172 in Acute Stroke Treatment subgroups.

DWI pattern	Etiology	OR	p-value	95%CI
Negative	LAA	1.00		
	LC	8.87	<0.001	4.03–19.52
	Undet	4.01	<0.001	1.84–8.7
	CE	2.46	0.029	1.1–5.49
	Other	2.04	0.13	0.80–5.24
Cortico-subcortical	LAA	1.00		
	CE	3.24	<0.001	1.9–5.52
	Undet	2.1	0.01	1.18–3.52
	Other	1.32	0.42	0.66–2.62
	LC	Empty		
Cortical	LAA	1.00		
	Undet	3.32	0.016	1.25–8.81
	CE	2.29	0.18	0.83–6.28
	Other	1.43	0.57	0.4–5.07
	LC	Empty		
Subcortical >20mm	LAA	1.00		
	Undet	2.44	0.04	1.04–7.73
	Other	2.35	0.08	0.88–6.24
	CE	2.2	0.07	0.92–5.23
	LC	0.15	0.08	0.01–1.28
Subcortical <20 mm	Undet	1.00	1.00	
	LC	42.9	<0.001	22.7–81.1
	LAA	3.47	<0.001	1.76–6.85
	Other	2.75	0.009	1.28–5.89
	CE	1.18	0.65	0.56–2.46
SSCL	Undet	1.00		
	LAA	4.22	<0.001	2.61–6.8
	Other	1.08	0.145	0.56–2.08
	CE	0.65	0.14	0.37–1.15
	LC	Empty		
SLVT	Other	1.00		
	LAA	1.97	0.18	0.74–5.18
	CE	1.88	0.17	0.75–4.7
	Undet	1.57	0.67	0.56–3.67
	LC	Empty		
MAC	Undet	1.00		
	CE	2.08	0.18	0.7–6.18
	Other	1.92	0.33	0.5–7.29
	LAA	1.34	0.66	0.35–5.07
	LC	Empty		
MPC	CE	1.00		
	Other	3.61	0.004	1.5–8.7
	Undet	3.61	0.004	1.5–8.7
	LAA	3.52	0.003	1.54–8.03
	LC	Empty		
MBAC	LC	1.00		
	Other	9.25	<0.001	1.12–76.2
	Undet	7.15	0.059	0.92–55.2
	CE	6.75	0.068	0.86–52.4
	LC	2.68	0.39	0.27–26
MAP	LAA	1.00		
	Other	5.54	0.001	1.94–15.1
	CE	4.30	0.003	1.64–11.2
	Undet	3.32	0.016	1.25–8.8
	LC	Empty		

DWI: diffusion-weighted imaging; OR: *Odds Ratio*; 95%CI: 95% confidence interval; LAA: large artery- atherosclerosis; LC: lacunar stroke; CE: cardioembolism; Other: stroke of other determined cause; Undet: Stroke of an undetermined cause, because the stroke was cryptogenic, 2 or more causes were identified, or there was an incomplete evaluation; SSCL: scattered lesions in one vascular territory of less than 15 mm; SLVT: scattered lesions in one vascular territory and confluent lesions of 15 mm or above; MAC: Multiple lesions in multiple vascular territories of unilateral anterior circulation; MPC: multiple lesions in the posterior circulation; MBAC: multiple bilateral lesions on anterior circulation; MAP: multiple lesions in both anterior and posterior circulation.

Cardioembolic strokes (CE) were associated with cortico-subcortical lesions (OR 3.24; 95%CI 1.9–5.5), multiple lesions in the anterior and posterior vascular territories (OR 4.3; 95%CI 1.64–11.2) and with negative DWI results (OR 2.46; 95%CI 1.1–5.49).

Cryptogenic strokes (only 4.46% of total number of cryptogenic cases had incomplete etiological evaluation) were associated with normal negative DWI results (OR 4.1; 95%CI 1.84–8.69), cortical lesions (OR 3.3; 95%CI 1.25–8.8), subcortical strokes with a diameter ≥20 mm (OR 2.44; 95%CI 1.04–5.73), multiple lesions in the anterior and posterior vascular territories (OR 3.33; 95%CI 1.25–8.81) and finally with cortico-subcortical strokes (OR 2.01; 95%CI 1.1–3.52).

Lacunar strokes (LC) correlated with a subcortical lesion with diameter <20 mm (OR 42.9; 95%CI 22.7–81.1) and only negative DWI patterns (OR 8.87; 95%CI 4.03–19.52).

Finally, other etiologies correlated with multiple lesions in the anterior and posterior vascular territories (OR 5.54; 95%CI 1.94–15.1), MBAC (OR 9.25; 95%CI 1.12–76.2) and MPC (OR 3.61; 95%CI 1.5–8.7).

## DISCUSSION

In this study we found an association between DWI lesion patterns and the TOAST causative classification; these have been previously described by some authors, but their studies included a limited number of patients^12,13^, they were mainly retrospective, and did not include negative or normal DWI results^10,11^, which are very frequent in the ER^4,5^ especially in patients with low NIHSS^14^.

LAA strokes were associated with scattered lesions in one vascular territory, with small scatter distribution. This finding has been demonstrated in other studies^10–12^ and considered highly suggestive for this etiology^12^.

MPC pattern was also associated with LAA strokes, a pattern that could be explained by a single atherosclerotic lesion in one of the vertebral arteries which could originate emboli to multiple arteries, including the basilar, cerebellar bilateral branches, and posterior cerebral arteries, causing multiple stroke lesions in the posterior circulation^15^.

LAA could also present as subcortical DWI lesions with diameter <20 mm, a finding not described previously. However, Lee et al.^16^ found that in their experience more than 53% of atherosclerotic middle cerebral artery lesions were clinically manifested as lacunar strokes and 30% or more had a small, deep subcortical lesion on DWI; the reasons for these lesions is the probable occlusion of a deep perforator lenticular artery by an atherosclerotic plaque in a mayor intracranial artery.

CE strokes were associated with lesions in multiple anterior and posterior arterial territories and cortico-subcortical lesions as described before^6,10,11^. We also found CE to be associated with negative DWI results; this is probably related to the fact that in our cohort patients with CE arrived earlier to the ER (p<0.001), probably because of the more dramatic symptoms (NIHSS was higher than in other etiologies, p<0.001). Early arrival to the ER is one of the factors that are critical for the negative DWI studies^4^.

Strokes of undetermined etiologies were associated with negative DWI, a finding described previously^4^. We could not rule out that some of our cryptogenic AIS were stroke mimic, but in this group of cryptogenic strokes, 19 patients (15.5%) had a intracranial arterial occlusion in the symptomatic territory, 52 (36.6%) patients had a stroke detected in the follow-up imaging, and 3 (2.1%) had perfusion abnormalities when the RAPID program was applied. Additionally, this subtype of stroke etiology was associated with cortico-subcortical lesions, which had been described as typical of CE strokes. This finding could be explained by the inclusion in this group of 21 cases who had two possible etiologies: all of them had atrial fibrillation as well as other possible causes for their AIS. As a consequence, they were classified as AIS of undetermined etiology. Some of these AIS were probably the result of their atrial fibrillation and of their episode of CE. This is a limitation of the TOAST causative classification. In our study, sub-cortical lesions with a diameter ≥20 mm were associated with AIS of undetermined etiology; a similar result was published by Kang et al. for lesions in these locations but whose size was greater than 15 mm^10^. Finally strokes of undetermined etiologies were also associated with involvement of multiples territories in the anterior and posterior circulation, a frequent finding in cardio-embolic strokes. This could be explained by the inclusion of patients with covert cardioembolic strokes mainly caused by paroxysmal atrial fibrillation not detected by telemetry and in whom prolonged monitoring has been shown to provide a significant increase in the probability of detecting AF^17^.

Lacunar strokes correlated with subcortical lesions whose diameter was <20 mm and with negative DWI results, a finding previously demonstrated^10,11^. Lacunar strokes had 60% less chance of abnormal DWI evaluation, probably due to their small size^3,4^.

Finally other etiologies were associated with multiple ischemic lesions in both anterior, MAP, and MBAC circulations. Of the patients with other etiologies, 22 (1.9%) had cancer-associated ischemic strokes and 22 (1.9%) had coagulopathies associated with the ischemic stroke; both groups of diseases had been shown to produce ischemia in multiple brain arterial territories^15,18^.

We can summarize our findings as follows: when there is an AIS with negative DWI the most likely etiology is cryptogenic and lacunar, unless, the patient has a high NIHSS in which case CE embolism could be the cause. Scattered lesions in one vascular territory should rise suspicion for LAA disease. Cortico-subcortical lesions are associated to CE etiology, as it is also the presence of multiple lesions in the anterior and posterior vascular territories. In this last group, cancer and coagulopathies could also be the etiology of this stroke. Finally, cryptogenic strokes can present with multiple DWI patterns.

Our study has several strengths: it included a large number of consecutive patients evaluated early in their evolution with DWI, with frequent addition of thin coronal sections for those cases with negative DWI, and with a few patients (less than 5%) with incomplete etiological studies.

Our study also has several important limitations, the main one is that it is a single-center experience in which we cannot rule out that some cases with negative DWI evaluations could correspond to stroke mimics. Also, DWI evaluations were performed on MRI with different field strength, which can influence the accuracy of DWI imaging. We also could not rule out the influence of the attending neurologist on the TOAST classification based on the DWI imaging. Furthermore, we did not study inter-observer agreement on the interpretation of DWI, and finally some DWI patterns could be associated with multiple etiologies. In conclusion we found an association between stroke subtypes by TOAST and the DWI lesion patterns.
